# Acute effects of a thermogenic nutritional supplement on cycling time to exhaustion and muscular strength in college-aged men

**DOI:** 10.1186/1550-2783-6-15

**Published:** 2009-07-13

**Authors:** Ashley A Walter, Trent J Herda, Eric D Ryan, Pablo B Costa, Katherine M Hoge, Travis W Beck, Jeffery R Stout, Joel T Cramer

**Affiliations:** 1 Biophysics Laboratory, Department of Health and Exercise Science, University of Oklahoma, Norman, Oklahoma, USA

## Abstract

**Background:**

The purpose of the present study was to examine the acute effects of a thermogenic nutritional supplement containing caffeine, capsaicin, bioperine, and niacin on muscular strength and endurance performance.

**Methods:**

Twenty recreationally-active men (mean ± SD age = 21.5 ± 1.4 years; stature = 178.2 ± 6.3 cm; mass = 76.5 ± 9.9 kg; VO_2 PEAK _= 3.05 ± 0.59 L/min^-1^) volunteered to participate in this randomized, double-blinded, placebo-controlled, cross-over study. All testing took place over a three-week period, with each of the 3 laboratory visits separated by 7 days (± 2 hours). During the initial visit, a graded exercise test was performed on a Lode Corival cycle ergometer (Lode, Groningen, Netherlands) until exhaustion (increase of 25 W every 2 min) to determine the maximum power output (W) at the VO_2 PEAK _(Parvo Medics TrueOne^® ^2400 Metabolic Measurement System, Sandy, Utah). In addition, one-repetition maximum (1-RM) strength was assessed using the bench press (BP) and leg press (LP) exercises. During visits 2 and 3, the subjects were asked to consume a capsule containing either the active supplement (200 mg caffeine, 33.34 mg capsaicin, 5 mg bioperine, and 20 mg niacin) or the placebo (175 mg of calcium carbonate, 160 mg of microcrystalline cellulose, 5 mg of stearic acid, and 5 mg of magnesium stearate in an identical capsule) 30 min prior to the testing. Testing included a time-to-exhaustion (TTE) ride on a cycle ergometer at 80% of the previously-determined power output at VO_2 PEAK _followed by 1-RM LP and BP tests.

**Results:**

There were no differences (*p *> 0.05) between the active and placebo trials for BP, LP, or TTE. However, for the BP and LP scores, the baseline values (visit 1) were less than the values recorded during visits 2 and 3 (*p *≤ 0.05).

**Conclusion:**

Our findings indicated that the active supplement containing caffeine, capsaicin, bioperine, and niacin did not alter muscular strength or cycling endurance when compared to a placebo trial. The lack of increases in BP and LP strength and cycle ergometry endurance elicited by this supplement may have been related to the relatively small dose of caffeine, the high intensity of exercise, the untrained status of the participants, and/or the potential for caffeine and capsaicin to increase carbohydrate oxidation.

## Introduction

The combination of nutritional supplements, such as caffeine and capsaicin, are commonly used as thermogenic aids to improve metabolism and performance [1-6]. Caffeine is sometimes consumed to enhance performance, whether that is athletic [1-5], cognitive [7,8], or immunological [9]. Extensive research has reported caffeine as a metabolic stimulant [6]. Capsaicin, the pungent component of hot red peppers, has been reported to evoke similar effects as caffeine [10-12]. In fact, the combination of caffeine, capsaicin, niacin, and bioperine has been reported to stimulate thermogenesis (i.e., burn more calories) when compared to a placebo [13]. Ryan et al. [13] reported that this particular combination of ingredients may be useful in maintaining a negative energy balance by increasing resting and low intensity energy expenditure. Therefore, there are limited data suggesting that the combination of caffeine, capsaicin, niacin, and bioperine may elicit metabolic adaptations to enhance exercise performance as well as resting energy expenditure.

## Background

Caffeine is among the most widely used drugs in the world and can be found in many foods including soft drinks, coffee, tea, and chocolate [14-17]. Caffeine has been shown to enhance exercise performance [18,19]. However, most previous studies have examined the effects of caffeine or caffeine-containing supplements on energy expenditure [13,20-22] or endurance performance [2,4,5,8,14,17,23-29]. It has been suggested that caffeine may augment catecholamine concentrations [30-32], potentiate calcium release from the sarcoplasmic reticulum in rodents and amphibians [33-37], and increase levels of muscle activation [15,38]. Therefore, potential mechanisms exist for caffeine to affect strength as well as endurance exercise performance. Indeed, several studies have reported improvements in aerobic running [23,24,27], cycling [4,5,8,26,29], and swimming [25] performance after caffeine supplementation. However, conflicting evidence exists regarding the effects of caffeine on anaerobic performance [7,39-42]. Beck et al. [39] administered a caffeine-containing supplement and demonstrated increases in bench press strength, but no changes in bench press endurance, leg extension strength or endurance, or power output during the Wingate test. Kalmar and Cafarelli [15] reported caffeine-induced increases in isometric leg extensor strength and endurance [15], whereas Astornio et al. [43] did not find improvements in leg press strength after caffeine supplementation. Make note, however, that the ergogenic potential of caffeine may be related to the training status of individuals [8,14,24,25,29,30,44], the amount of caffeine consumed [5,8,45,46], and the intensity and duration of exercise [4,5,7,19,47,48].

Capsaicin, the pungent component of hot red peppers, has been reported to evoke similar effects as caffeine. Watanabe et al. [10] suggested that the primary mechanism of capsaicin is the β-adrenergic stimulation that induces catecholamine release. Kawada et al. [49] reported an increase and then decrease in the respiratory quotient (RQ) after capsaicin ingestion, suggesting an increase in carbohydrate and then fat mobilization. Kim et al. [50] and Ohnuki et al. [51] reported increases in lypolysis after ingesting 10 mg·kg^-1 ^body weight of capsaicin in mice. The authors suggested that the increases were due to the glycogen sparing effect of capsaicin during exercise, while fatty acids were used as fuel. Additionally, Yoshioka et al. [11,12] suggested that the capsaicin-induced increases in energy expenditure were due to sympathetic nervous system activation, which can influence fat oxidation and catecholamine release. This hypothesis has been supported by Kim et al. [50] and Oh et al. [52,53]. In contrast, Lim et al. [54] reported the opposite effect (i.e. carbohydrate oxidation), such that the RQ was higher after ingesting capsaicin when compared to a control. The authors [54] suggested that endurance performance may have been limited by exhausting the glycogen stores, rather than utilizing fat as fuel.

In addition to caffeine and capsaicin, bioperine (black pepper extract) and niacin (vitamin B_3_) may also enhance thermogenesis when taken as a nutritional supplement. Bioperine, the thermogenic ingredient in black pepper, has been reported to increase the metabolism in rats [55,56]. Furthermore, niacin has been used in medications to help lower cholesterol by increasing fatty acid mobilization and may act as a peripheral vasodilator [57]. Thus, the combination of various nutritional supplements that may enhance the metabolic rate, such as caffeine, capsaicin, bioperine, and niacin, may also result in acute improvements in performance. Additionally, the combination of ingredients in this nutritional supplement may have a synergistic effect because the caffeine and capsaicin have similar properties, in addition to the niacin which would increase blood flow and fatty acid mobilization. Therefore, the purpose of the present study was to examine the acute effects of a thermogenic nutritional supplement containing caffeine, capsaicin, bioperine, and niacin on muscular strength and endurance performance.

## Methods

### Subjects

Twenty healthy men (mean age ± SD; 21.5 ± 1.4 years; height: 178.2 ± 6.3 cm; weight: 76.5 ± 9.9 kg; VO_2 PEAK_: 3.05 ± 0.59 L/min^-1^) volunteered for this investigation. Each subject completed a pre-exercise health status questionnaire and signed a written informed consent document. This study was approved by the University of Oklahoma Institutional Review Board for human subjects research. All of the subjects reported being recreationally active (5.4 ± 3.02 hours of exercise per week), however, none of the subjects were competitive athletes. In addition, none of the subjects reported or exhibited any of the following: (a) a history of medical or surgical events that might have significantly affected the study outcome, including cardiovascular disease or metabolic, renal, hepatic, or musculoskeletal disorders; (b) use of any medications that might have significantly affected the study outcome; (c) use of nutritional supplements (e.g., creatine, protein drinks, amino acids, or vitamins) in the 9 weeks prior to this study; or (d) participation in another clinical trial or ingestion of another investigational product within 30 days prior to this study.

### Study Design

This study used a randomized, double-blind, placebo-controlled, cross-over design. All testing took place over a three-week period, with each laboratory visit separated by 7 days (± 2 hours). During the first week, participants completed the baseline testing, which included a graded exercise test (GXT) on a cycle ergometer to determine maximal oxygen consumption rate (VO_2 PEAK_) and one-repetition maximums (1-RM) for the leg press (LP) and bench press (BP) to assess muscle strength. During weeks 2 and 3, the subjects were asked to consume a capsule containing either the active supplement or the placebo (in random order) 30 min prior to the testing, which included a time-to-exhaustion (TTE) ride on a cycle ergometer at 80% of the previously-determined VO_2 PEAK _followed by 1-RM LP and BP tests.

### Supplementation Protocol

For the final two laboratory visits (weeks 2 and 3), subjects received either the supplement or the placebo in random order. The thermogenic pepper blend (TPB) supplement contained 200 mg of caffeine, 33.34 mg of capsicum extract (0.67 mg of capsaicin at 100,000 scoville heat units), 20 mg of niacin, and 5 mg of bioperine (black pepper extract). The placebo (PL) contained 175 mg of calcium carbonate, 160 mg of microcrystalline cellulose, 5 mg of stearic acid, and 5 mg of magnesium stearate. Both the TPB and PL capsules were dark, opaque, and similar in appearance to maintain the double blind nature of the experiment. In addition, 3^rd ^party random laboratory testing (Nutra Manufacturing Inc., Greenville, SC) was performed to confirm that the ingredients in the TPB and PL capsules were within ± 5% of the ingredients claimed above.

### Graded Exercise Test Protocol

The GXT was completed on an electronically-braked cycle ergometer (Lode, Groningen, Netherlands). Prior to any bike tests, participants' seat height was measured and recorded for consistency between trials. Participants stood next to the bike to estimate proper seat height (greater trohcanter), then mounted to ensure there was a slight bend at the knee at the bottom of the pedal stroke, not full or hyperextension. Once the seat height was set and comfortable, the foot straps were secured and were used to prevent the participant's feet from slipping off the pedals during the test. After a five minute warm-up at 50 W, the workload increased an additional 25 W every two minutes. Participants were encouraged to maintain 70 rpm, but the test was terminated when the participant could no longer maintain 60 rpm (volitional exhaustion). Each participant's rating of perceived exertion (RPE) was also recorded during every stage using a standard Borg scale [58]. A true VO_2 PEAK _was determined if three of the five indicators were met during the test according to the American College of Sports Medicine Guidelines [59].

### Determination of Maximal Oxygen Consumption Rate

Respiratory gases were collected and monitored using a metabolic cart (Parvo Medics TrueOne^® ^2400 Metabolic Measurement System, Sandy, Utah). The metabolic cart was calibrated prior to each test with room air and standard gases of known volume and concentration for the O_2 _and CO_2 _analyzers. Flowmeter calibration was also performed prior to each GXT. Respiratory gases were collected by use of a two-way rebreathing valve (Hans-Rudolph Inc., Shawnee, Kansas) and mouthpiece attached to headgear, which held them in place. Participants wore a nose clip to ensure that breathing occurred entirely through the mouth. O_2 _and CO_2 _were analyzed through a sampling line after the gasses passed through a heated pneumotach and mixing chamber. The metabolic cart software reported the values as ventilated oxygen and carbon dioxide (VO_2 _and VCO_2_, respectively) and calculated VO_2 PEAK _automatically.

### Muscular Strength Assessment

Subjects performed tests to determine 1-RM for the incline leg press (LP) and bench press (BP) exercises. The LP exercise was performed using a plate-loaded hip sled with a 45° incline (Paramount Fitness Corp., Los Angeles, California). Subjects sat in the seat with their back flat against the backrest and were instructed to grasp the handles of the device tightly to avoid the buttocks losing contact with the seat during the exercise. Subjects placed their feet in the middle of the platform at shoulder's width apart, and this foot position remained constant for all the subsequent leg press tests. Subjects were instructed to lower the platform until the legs reached 90° of flexion at which point they were instructed to fully extend the legs (i.e., 0° of leg flexion). The BP exercise was performed on a standard free-weight bench (TuffStuff, Pomona, California) with an Olympic bar. After receiving a lift-off from a spotter, subjects lowered the bar to their chest, paused briefly, and then pressed the bar to full extension of the forearms. If a repetition for either the LP or BP exercises did not meet the aforementioned criteria, it was not counted, and another attempt was allowed after a 2-min rest period. For both the LP and BP exercises, 1-RM strength was determined by applying progressively heavier loads until the subject could not complete a repetition through the full range of motion. If an attempt was failed and a lower weight not attempted, additional trials were performed with the lighter load, and successive increases in weight until the 1-RM was determined, which was usually achieved within 5 trials. Two minutes of rest were allowed between trials [60].

### Time to Exhaustion

During the final two laboratory visits (weeks 2 and 3), TTE was also measured on the same cycle ergometer as the GXTs. The seat height was adjusted to the previously-recorded height. The test began with a warm-up consisting of 5 min of cycling at 70 rpm against a resistance of 50 W. Following the warm-up, participants cycled at a workload associated with 80% of the previously-determined VO_2 PEAK_. Participants were instructed to maintain 70 rpm, but the test was terminated when the participant could no longer maintain 60 rpm (volitional exhaustion). Participants were provided verbal encouragement throughout the duration of the test. Time was measured using a digital stopwatch and was recorded in seconds. RPE was also assessed every minute throughout the duration the test.

### Statistical Analyses

Two separate one-way repeated measures analyses of variance (ANOVAs) (baseline vs. trial 1 vs. trial 2) were calculated for the 1-RM LP and BP scores. When appropriate, post hoc pair-wise comparisons with Bonferroni adjustments were completed. In addition, paired-samples t-tests were used to compare the mean TTE and RPE values between weeks 2 and 3. Prior to all statistical analyses, the alpha level was set at *p *≤ *0*.05 to determine statistical significance. Data were analyzed using SPSS for Windows version 14.0 (SPSS Inc., Chicago, IL).

## Results

There were no differences (*p *> 0.05) between the TPB and PL trials for BP, LP, TTE, or RPE. However, for the BP and LP scores, the baseline values were less than the TPB and PL values (*p *≤ 0.05) (Table 1).

**Table 1:** Mean(SE) values for bench press and leg press 1-RM, time-to-exhaustion, and rating of perceived exertion

	**Bench Press 1-RM** (*kg*)	**Leg Press 1-RM** (*kg*)	**Time to Exhaustion** (*s*)	**Rating of Perceived Exertion**
*Baseline*	80.80 (5.21)	215.00 (12.45)	---- ----	---- ----
*Placebo*	82.39* (5.08)	225.80* (12.54)	602.23 (51.78)	15.80 (0.25)
*Supplement*	82.73* (5.36)	224.04* (12.93)	633.19 (52.88)	15.70 (0.22)

*denotes a significant (*p *≤ 0.05) difference from baseline

## Discussion

Our findings indicated that the TPB supplement containing caffeine, capsaicin (red pepper extract), bioperine (black pepper extract) and niacin did not significantly (*p *≤ 0.05) alter the BP or LP 1-RMs, TTE at 80% VO_2 PEAK_, or RPE during the TTE test. Even though the TTE was approximately 5% greater for the TPB supplement compared to the PL (Table 1), this finding did not reach statistical significance (*p *= 0.403). This experiment was unique in that strength- and endurance-related variables were examined in response to a combination of caffeine and capsaicin, both of which have been studied separately [12,14,43,48,54]. We are aware of only a few other studies that have examined the effects of a similar blend of supplements on exercise performance and/or energy expenditure [11,13,20,61]. For example, Yoshioka and colleagues [11] reported higher energy expenditure after a meal containing red pepper and caffeine when compared to a control meal. Similarly in obese individuals, capsaicin and caffeine (among other ingredients) enhanced resting metabolic rate by 90 kJ, which suggested that these supplements exhibited a thermogenic effect at rest [20]. In addition, Ryan et al. [13] indicated that a caffeine- and capsaicin-containing supplement increased energy expenditure in healthy sedentary subjects before, during, and after 1 hour of light aerobic exercise. Therefore, these results collectively suggested that the potential thermogenic benefits of supplements containing caffeine and capsaicin may be more realized at rest (5,19,22) and during light aerobic exercise (19) than during anaerobic (1-RMs) and high-intensity aerobic (TTE at 80% VO_2 PEAK_) exercises as indicated by the results of the present study.

Several studies have examined the ergogenic benefits of caffeine supplementation as indicated by several thorough literature reviews [3,5,16,18,41,62-64]. Most of this literature focuses on the effects of caffeine supplementation on relatively low- to moderate-intensity endurance performance [2,5,14,16,17,62]. Fewer studies have reported changes in muscle strength after caffeine supplementation [15,39,43]. Beck et al. [39] and Kalmar and Cafarelli [15] reported caffeine-induced increases in 1-RM bench press strength and voluntary muscle activation, respectively. However, Astorino et al. [43] and Beck et al. [39] also reported no caffeine-related changes in 1-RM leg press and leg extension exercises, respectively. In addition, Bond et al. [42] and Jacobson et al. [45] reported no changes in isokinetic strength of the leg extensors and flexors after various doses of caffeine. It has been suggested that calcium is more readily available for release from the sarcoplasmic reticulum after caffeine administration in rodents and frogs [33-37]. In addition, caffeine may alter the activation thresholds of motor neurons, resulting in increased motor unit firing and activation of more muscle [32]. In the present study, however, there was only 200 mg of caffeine in the TPB supplement, which is less than most caffeine doses administered in previous studies [15,32,42,43,45,65,66]. Therefore, the lack of observed differences in the present study may have been due to the relatively small dose of caffeine in the TPB supplement, since the ergogenic effects of both caffeine [2,17,67] and capsaicin [22,52] may be dose-dependent.

Although the effects of caffeine on strength measures are relatively inconclusive, studies have reported improvements in endurance performance after caffeine supplementation [2,5,14,16,17,62]. For example, increases in TTE with caffeine supplementation in well-trained subjects have been demonstrated [8,14,24,25,29,44]. However, other studies that have tested untrained subjects [26,65,68] have found no changes in TTE after caffeine ingestion. Arguments have been made that the subjects' initial training status is the primary limiting factor for TTE performance [65], especially at relatively high workloads, such as those used in the present study. In support of this hypothesis, Hogervorst et al. [8] reported an 84% increase in TTE after a 2.5-h bout of cycling at 60% of the VO_2MAX _with well-trained cyclists after only 100 mg of caffeine was taken at several intervals. Therefore, the ergogenic effects of lower doses of caffeine may be more profound in trained individuals at lower-intensity, longer-duration endurance events. Since the participants in the present study were untrained and the exercise intensity was relatively high (80% VO_2 PEAK_), the caffeine-induced improvements in performance may have been less evident.

As with many ergogenic aids, the amount of caffeine supplementation may be proportional to the magnitude of performance improvements. Jenkins et al[5] reported increases in cycling performance with as low as 2 mg of caffeine per kilogram of body mass (mg·kg^-1^) in trained cyclists. In contrast, Pasman et al. [29] reported no dose-response relationship between caffeine consumption and TTE at 80% of the maximal cycling wattage (W) with 5, 9, and 13 mg·kg^-1^. However, even the minimal dose administered by Pasman et al. [29] was approximately 360 mg (5 mg·kg^-1 ^× mean body mass of 72 kg). The absolute caffeine dose administered in the present study was only 200 mg (~2.6 mg·kg^-1^), which may have limited the potential ergogenic effects that are often observed with caffeine consumption. Nevertheless, our findings were similar to those of Bell et al. [65], which used a workload at 85% of the VO_2MAX _and reported mean TTE values of 14.4 and 12.6 min for the caffeine (5 mg·kg^-1^) and placebo trials, respectively. The results of the present study indicated that the TTE for the TPB supplement was 5% greater than the PL trial (Table 1), although this finding was not statistically significant (*p *= 0.403). Therefore, because the caffeine dose administered in the present study was lower than what has been used in previous studies [15,32,42,43,45,65,66], the consequent ergogenic effects of caffeine may also have been limited.

The combination of caffeine and capsaicin supplements may potentially yield synergistic, ergogenic effects. For example, the elevation of plasma catecholamines after caffeine or capsaicin ingestion have previously resulted in increased lypolysis [14,17,44] and decreased carbohydrate utilization [69]. Yoshioka et al. [12] suggested that the primary mechanism of capsaicin is the β-adrenergic stimulation that induces thermogenesis. Recently, Lim et al. [70] reported a glycogen-sparing effect in rats during 2 h of treadmill running with a consequent increase in lypolysis after ingesting 6 mg·kg^-1 ^of capsaicin. However, a more recent study by Lim et al. [54] reported that 10 g of red peppers (containing capsaicin) taken before exercise increased carbohydrate oxidation, which the authors suggested could limit endurance performance by exhausting glycogen stores. These findings [54] may, in part, explain the results of the present study, which found no differences in cycling endurance time between the TPB and PL trials. Additional ingredients in the TPB supplement included black pepper extract (i.e., bioperine), which is purported to have same metabolic effects as capsaicin. It is possible that the combined effects of caffeine, capsaicin, bioperine, and niacin may be most evident at higher doses during longer duration, lower intensity endurance exercises – particularly in trained individuals [8,24]. Future research is necessary to examine the potential dose-response mechanisms for the TPB supplement ingredients during a range of exercise intensities.

An interesting outcome was that the BP and LP 1-RM values at baseline were less than the 1-RM values recorded for the TPB and PL trials (Table 1). These results suggested that the participants experienced a learning effect from the baseline trial to the TPB or PL trials [71]. Hyllegard, Mood, and Morrow [71] recommend using a baseline familiarization or "learning" trial to overcome the confounding influences of the learning effect. Therefore, the inclusion of the baseline measurement in the present study may have been helpful to avoid the learning effect for the 1-RM scores. In addition, the average TTE was approximately 5% greater for the TPB trial than the PL trial (Table 1). Perhaps the relatively high variability in TTE scores (coefficient of variation = 37.5%) may have prevented this difference from reaching statistical significance.

## Conclusion

Overall, the results of the present study indicated that the TPB supplement containing 200 mg of caffeine, 33.34 mg of capsicum extract, 20 mg of niacin, and 5 mg of bioperine did not improve the 1-RM scores for the BP or LP exercises, TTE at 80% VO_2 PEAK_, or RPE during the TTE test. Even though the TTE for the TPB supplement was 5% greater than the PL trial (Table 1), this finding did not reach statistical significance (*p *= 0.403). The lack of observed ergogenic effects may have been related to a combination of factors including: (a) the dose of caffeine was too low, (b) the exercise intensity was too high for a metabolic-enhancing supplement like TPB, (c) the participants were not well-trained, and/or (d) the caffeine and capsaicin may have increased carbohydrate oxidation (as opposed to the glycogen sparing effect [17]), which may have counteracted any potential ergogenic effects of the TPB. Although these findings are useful for understanding the TPB supplement, future studies should consider these factors when designing studies to examine the efficacy of metabolic/thermogenic nutritional supplements.

## Competing interests

The authors declare that they have no competing interests.

## Authors' contributions

AAW was the primary author of the manuscript and played an important role in data collection and assessment. TJH, EDR, PBC, and KMH played an important role in data collection and manuscript preparation. JRS and TWB played an important role in study design and manuscript preparation. JTC was the senior author and played an important role in the grant procurement, study design, data analysis and interpretation, and manuscript preparation. All authors have read and approved the final manuscript.
